# Aggregation as bacterial inclusion bodies does not imply inactivation of enzymes and fluorescent proteins

**DOI:** 10.1186/1475-2859-4-27

**Published:** 2005-09-12

**Authors:** Elena García-Fruitós, Nuria González-Montalbán, Montse Morell, Andrea Vera, Rosa María Ferraz, Anna Arís, Salvador Ventura, Antonio Villaverde

**Affiliations:** 1 Institut de Biotecnologia i de Biomedicina, Universitat Autònoma de Barcelona, Bellaterra, 08193 Barcelona, Spain; 2 Departament de Genètica i de Microbiologia, Universitat Autònoma de Barcelona, Bellaterra, 08193 Barcelona, Spain; 3 Departament de Bioloquímica i de Biologia Molecular, Universitat Autònoma de Barcelona, Bellaterra, 08193 Barcelona, Spain

## Abstract

**Background:**

Many enzymes of industrial interest are not in the market since they are bio-produced as bacterial inclusion bodies, believed to be biologically inert aggregates of insoluble protein.

**Results:**

By using two structurally and functionally different model enzymes and two fluorescent proteins we show that physiological aggregation in bacteria might only result in a moderate loss of biological activity and that inclusion bodies can be used in reaction mixtures for efficient catalysis.

**Conclusion:**

This observation offers promising possibilities for the exploration of inclusion bodies as catalysts for industrial purposes, without any previous protein-refolding step.

## Background

Protein misfolding is a common event during bacterial over-expression of recombinant genes [1]. The aggregation of insoluble polypeptide chains as inclusion bodies has seriously restricted the spectrum of proteins marketed by the biotechnology industry. Being widespreadly believed that inclusion body proteins are biologically inactive and therefore useless in bioprocesses, many aggregation-prone products have been disregarded for commercialisation. Protein solubility can be tailored by either process [2] or protein [3] engineering, although most efforts have been addressed to minimize inclusion body formation by co-production of folding modulators [4], or to refold purified inclusion body proteins by chemical denaturation followed by refolding procedures [5]. Both strategies need to be adapted to particular protein species and they render largely variable results regarding the final soluble protein yield.

Interestingly, independent reports have noted enzymatic activity associated to inclusion bodies formed by recombinant enzymes [6-8], but the extent of these side-observations has been never quantified and its biological and biotechnological relevance remained unexplored. In this work, we have quantitatively explored the biological activity of inclusion body recombinant proteins and their potential use for bioprocesses in the aggregated form.

## Results

**Figure 1:**
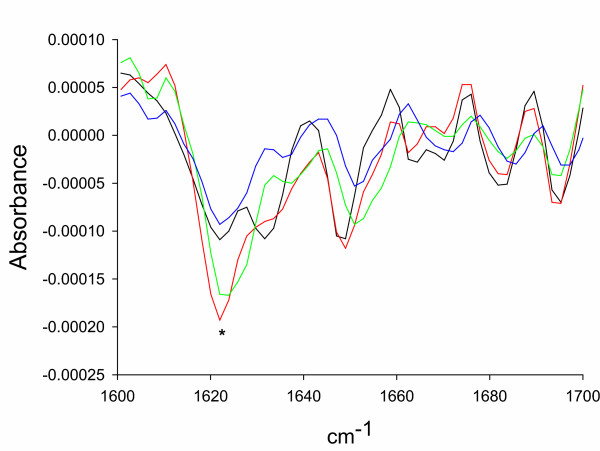
FTIR spectra of inclusion bodies formed by either VP1LAC (black), hDHFR (green), VP1GFP (red) or Aβ42(F19D)-BFP (blue) in the amide I region [9]. The asterisk labels the peak indicative of extended inter-molecular β-sheet structures in bacterial inclusion bodies.

**Figure 2:**
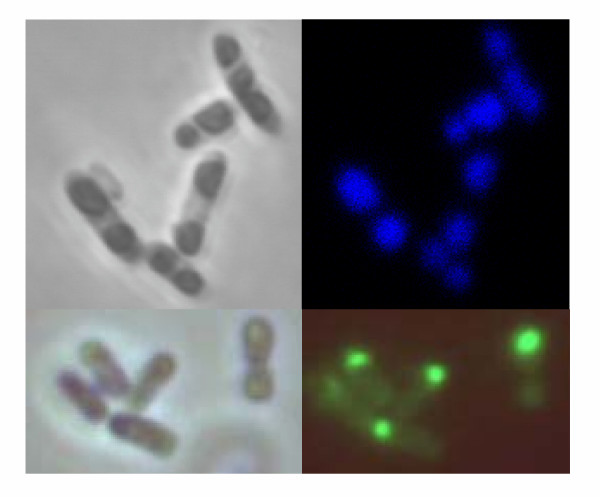
Optical micrographs of Aβ42(F19D)-BFP (top) and VP1GFP (bottom) inclusion bodies by phase contrast (left) and fluorescent microscopy (right).

To determine the occurrence of active protein in inclusion bodies we analysed those formed upon overproduction of the wild-type human dihydrofolate reductase (hDHFR) and an engineered *E. coli *β-galactosidase fused to the aggregation-prone foot-and-mouth disease virus (FMDV) VP1 capsid protein (VP1LAC). In addition, we explored fluorescence emission of green and blue fluorescent proteins (GFP and BFP respectively) fused to different aggregating polypeptides, namely the FMDV VP1 and a point mutant of the human Aβ-amyloid peptide (Aβ(F19D)), by comparing specific fluorescence emission of protein in the soluble cell fraction and purified inclusion bodies. Upon overproduction, all these proteins form cytoplasmic inclusion bodies in *E. coli*, the fraction of the aggregated protein ranging between 28 and 88 % of the total recombinant production (Table 1). Surprisingly, both enzymatic activity and specific fluorescence of inclusion body proteins were unexpectedly high (Table 1), ranging from 6 to 166 % of that of their counterparts occurring in the soluble cell fraction. This fact indicates that protein inactivation mediated by *in vivo *aggregation is only moderate. In addition, it is shown that protein packaging as bacterial inclusion bodies into inter-molecular β-sheet architecture (characterized by the presence of a peak around 1620 cm^-1 ^that dominates the FTIR spectrum in the amide I region) [9,10] in these model proteins (Figure 1) is compatible with the functionality of enzyme active sites and fluorophores. In this context, VP1GFP and Aβ42(F19D)-BFP inclusion bodies are noticeably fluorescent inside the producing cells (Figure 2).

**Table 1:** Enzymatic activity or fluorescence of inclusion bodies produced in *E. coli*

Construct name	Reference	Functional protein	Fraction of inclusion body protein (range, %) ^a^	Aggregating domain or protein (all in the N-terminal position)	Specific activity or emission ^b ^(enzymatic units/mg or fluorescence units/mg)		Activity of the inclusion body fraction relative to that of soluble protein (%) ^c^
						
					Soluble protein	Inclusion bodies	
VP1LAC	This work and [9]	*E. coli *β-galactosidase	35.6–45.9	FMDV VP1 capsid protein	698.3 ± 153.0	1162.5 ± 256.0	166.4
hDHFR	[25]	Human dihydrofolate reductase	28.4–36.8	none	8.0 10^-2 ^± 2.6 10^-2^	4.7 10^-3 ^± 0.9 10^-3^	5.9
VP1GFP	This work	Green fluorescent protein	82.5–88.4	FMDV VP1 capsid protein	359.5 ± 66.0	70.4 ± 10.1	19.5
Aβ42(F19D)-BFP	[26]	Blue fluorescent protein	61.4–65.3	Aβ42(F19D)	118.1 ± 10.2	36.3 ± 2.2	30.7

^a ^The percentage of protein found in inclusion bodies relative to the total intracellular amount of recombinant protein. Values were determined from different samples taken at 3 and 5 h after triggering recombinant gene expression.

^b ^These values were determined in samples taken between 3 and 5 h after triggering recombinant gene expression.

^c ^Specific activity or fluorescence emission of inclusion bodies relative to the values determined for the soluble counterpart fraction. Protein amounts were determined by Western blot analysis as described and enzymatic assays performed by conventional procedures. Excitation wavelengths were 450 nm for VP1GFP and 360 nm for Aβ42(F19D)-BFP.

**Figure 3:**
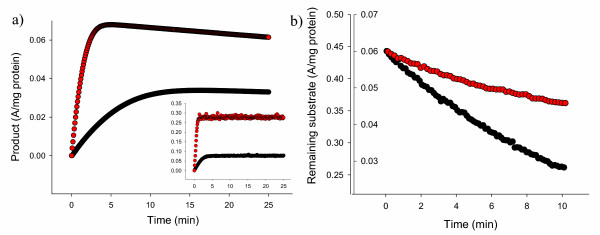
A) Product formed by soluble (black symbols) or inclusion body (red symbols) VP1LAC through ONPG hydrolysis as determined at 414 nm. Very coincident results have been obtained by using CPRG as alternative substrate (see the small panel), whose hydrolysis product was determined at 540 nm. B) Conversion of NADPH into NADP^+ ^associated to tetrahydrofolate formation mediated by soluble (black symbols, left scale) and inclusion body (red symbols, right scale) hDHFR. Absorbance was determined at 340 nm.

We wondered if active inclusion bodies could be then used in suspension as efficient catalysts for bioprocesses. If so, the straightforward use of these particles, that in addition are easily removable from the reaction mixture once the reaction is completed by low speed centrifugation, would be a convenient alternative to in vitro protein refolding before use, a complex procedure for which efficiencies are highly variable but in general low [5]. The enzymatic activity of soluble and inclusion body versions of both VP1LAC and hDHFR was then monitored in reaction mixtures. As observed (Figure 3A and 3B), inclusion body-embedded enzymes performed very efficiently as catalysts of enzymatic reactions. Substrate hydrolysis mediated by the insoluble form of VP1LAC was significantly faster than that mediated by the same amount of the soluble version (Figure 3A), while substrate processing by hDHFR was slower when driven from inclusion bodies but still important (Figure 3B). These observations are nicely compatible with the specific activities displayed by both versions of these proteins (Table 1).

## Discussion

The quantitative similarity between protein activity in the soluble cell fraction and that of the aggregated forms of both enzymes and fluorescent proteins (Table 1) demonstrates that physiological aggregation as inclusion bodies does not necessarily split protein population into active and inactive fractions. Probably, protein solubility (observed as the occurrence in the soluble cell fraction) does not necessarily indicate the acquisition of a correctly folded and thus active structure. In this context, soluble micro-aggregates have been described [11] and recently characterized in detail [12]. The non complete coincidence between solubility and folding has been previously indicated by exhaustive mutational analysis of model proteins [13], showing that the genetic determinants of protein aggregation and misfolding are not coincident. In this way, natively unfolded proteins are unstructured but soluble [14]. Therefore, determinations of GFP-fusions solubility by using fluorescence as reporter [15] could have eventually been indicative of folding-misfolding extend rather than solubility-insolubility, since inclusion bodies formed by GFP fusions can be highly fluorescent (Figure 2). Furthermore, solubility does not appear to be an all-or-nothing attribute and polypeptide chains might exhibit a continuum of folding states in both soluble and insoluble cell fractions, between which they are dynamically transferred with the assistance of cellular folding modulators [16]. In this context, the occurrence and evolution of 'soluble' aggregates in bacteria (namely misfolded species occurring in the soluble cell fraction and presumably inactive) [12] could explain the variable specific activity observed in the soluble cell fraction of bacteria producing recombinant β-galactosidases [17].

Inversely, our results prove a major occurrence of native or native-like protein in inclusion bodies. In fact, deposition as inclusion bodies might even result in the enrichment of active species as suggested by the specific activity (166 % of that found in the soluble cell fraction; Table 1) and catalytic properties (Figure 3A) of VP1LAC inclusion bodies. This observation can be then again indirectly indicative of the presence of enzymatically inactive protein in the soluble cell fraction, since protein deposition is not expected to favour a correct folding.

Finally, although the existence of native-like structure in bacterial inclusion body proteins has been previously reported [18], here we demonstrate that this is not anecdotic but probably the architectonic nature of these kind of aggregates, as inclusion bodies formed by four structurally different proteins all display significantly high biological activity. Interestingly, the active and properly folded polypeptides in inclusion bodies coexist with a molecular β-sheet organization also manifest in all cases, although the extent of β-sheet structure and its coincidence with the biological activity of the aggregates cannot be quantitatively evaluated. Since is highly improbable that enzyme active sites involved in the intermolecular β-sheet structure could be themselves active, we suggest that enzymatic activity or fluorescence are supported by properly folded molecules or molecule segments. Aggregation, observed as protein deposition driven by intermolecular interactions between solvent-exposed hydrophobic patches [9] would not necessarily disturb the conformation of all protein domains, and the active site would be still functional if misfolded, aggregation-prone regions are located in a distant site of the polypeptide chain. Alternatively, properly folded and active molecules could coexist with β-sheet-enriched (inactive) versions of the same species, and both situations could in fact take place simultaneously in single aggregate units. Further structural and functional analysis would hopefully solve this issue.

From an applied point of view, inclusion bodies, being formed by sequence-specific interaction between homologous protein patches result in highly pure protein microparticles [9]. Since they are also porous and highly hydrated [19], efficient substrate diffusion would probably occur for most of the (or at least many) biotechnologically relevant aggregated enzymes, thus opening the possibility for a new industrial market of enzymatically active inclusion bodies.

## Conclusion

Results presented here prove that aggregation of recombinant proteins as bacterial inclusion bodies does not necessarily inactivate them, despite the enriched intermolecular β-sheet structure observed in those formed by the tested model proteins. The extent of protein activity varies depending on the specific protein, but even the lowest functional values observed are still high enough to consider the use of inclusion body enzymes in bioprocesses, without any previous refolding step. The eventual incorporation of inclusion bodies in industrial catalysis could represent an important conceptual shift in the biotechnology market.

## Methods

### Strain, plasmids and culture conditions

*E. coli *MC4100 [20] was used for all the experiments. Plasmids encoding hDHFR and Aβ42(F19D)-BFP have been previously described and appropriate references can be found in Table 1. Briefly, in the Aβ42(F19D)-BFP vector (6.7 Kb) the DNA sequence encoding the 42-mer Alzheimer's amyloid peptide, (bearing a Phe^19^→Asp mutation to reduce its *in vivo *aggregation rate), is fused upstream of the BFP gene and under the control of the T7 promoter, in a pET-28 based vector. In the product, the two protein sequences were separated by 12-mer linker stretch to provide flexibility to the fusion protein and limit steric constraints between domains. pTVP1LAC was constructed by moving the *Sal*I-*Nco*I VP1LAC fusion-encoding DNA segment (3.5 Kb) from pJVP1LAC (8.5 Kb) to the cloning vector pTRC99A [20]. The resulting pTVP1LAC construct (7.7 Kb) was used to direct the production of VP1LAC. The *lacZ *gene was further replaced there by an appropriate GFP-encoding DNA segment (0.7 Kb) through digestion with *EcoR*I and *BamH*I, rendering pTVP1GFP (5.5 Kb). All the production processes were performed in shaker-flask cultures growing at 37°C in LB rich medium [20] plus 100 μg/ml ampicillin for plasmid maintenance, and recombinant gene expression was induced when the OD_550 _reached 0.4, by adding 1 mM IPTG. Cell samples were taken at 3 and 5 h after induction of gene expression.

### Analysis of enzymatic activity

Culture samples of 2.5 ml were jacketed in ice, disrupted by sonication for 5 min at 50 W under 0.5 s cycles [21] and centrifuged at 4°C for 15 min at 15000 g. The supernatant was directly used for the analysis as the soluble cell fraction. Inclusion bodies were purified by a detergent-washing protocol as described [19] and used in suspension for activity analysis. β-Galactosidase activity of both soluble cell fraction and inclusion bodies of VP1LAC was determined in microplates as described [7,22] under continuous stirring at 250 rpm. Kinetic analysis of VP1LAC enzymatic activity was monitored in 120 μl reaction mixtures with either 2 mM ONPG (pH 8.4) or 2 mM CPRG (pH 7.0). The hDHFR activity was determined by incubating 50 μl of the protein sample and 850 μl of the appropriate assay buffer (0.1 M K_3_PO_4 _pH 7.4, 1 mM DTT, 0.5 M KCl, 1 mM EDTA and 20 mM ascorbic acid) for 10 minutes at room temperature. Then, 50 μl of 2 mM 7,8-dihidrofolate and 50 μl of 2 mM NADPH were added and hDHFR activity was recorded every 15 seconds during 4 minutes at 340 nm. Protein concentration in all the assays was adjusted between 2 and 3 μg/ml.

Fluorescence (at 510 nm for GFP and 460 nm for BFP) was recorded in a Perkin-Elmer 650-40 fluorescence spectrophotometer by using excitation wavelengths of 450 nm and 360 nm for GPF and BFP respectively. Fluorescence was measured in 1 ml samples using dilutions when necessary. Both enzymatic activities and fluorescence were determined in triplicate.

### Quantitative protein analysis

Samples of bacterial cultures (10 ml) were low-speed centrifuged (15 min at 12000 g) to harvest the cells. For protein quantification in soluble cell fractions, samples were resuspended in 400 μl of Z buffer without β-mercaptoethanol [23] with one tablet of protease inhibitor cocktail (Roche, ref. 1 836 170) per 10 ml buffer. Such mixtures, once jacketed in ice, were sonicated for 5 min (or longer when required to achieve a complete disruption) at 50 W under 0.5 s cycles as described [21], and centrifuged for 15 min at 12000 g. The supernatant was mixed with denaturing buffer at appropriate ratios [24]. For the determination of inclusion body protein, these structures were purified by repeated detergent washing as described [19] and resuspended in denaturing buffer [24]. After boiling for 20 min, appropriate sample volumes were loaded onto denaturing gels. For Western blot, polyclonal antibodies specific for each protein were used as previously described [17]. Dried blots were scanned at high resolution and bands quantified by using the Quantity One software from Bio Rad, by using appropriate protein dilutions of known concentration as controls. Determinations were always done within the linear range and they were used to calculate the specific activity values.

### Conformational analysis by FTIR spectroscopy

For FTIR spectroscopy analysis, purified inclusion bodies were dried for two hours in a Seepd-Vac system before analysis to reduce water interference in the infrared spectra. The FTIR spectrum of the dry samples was analysed directly in a Bruker Tensor FTIR spectrometer. All processing procedures were carried out so as to optimise the quality of the spectrum in the amide I region, between 1600 cm^-1 ^and 1700 cm^-1^. Second derivatives of the amide I band spectra were used to determine the frequencies at which the different spectral components were located. A general description of FTIR procedures can be found elsewhere [9,10].

## Abbreviations

BFP blue fluorescent protein

CPRG phenol red β-D-galactopyranoside

FMDV foot-and-mouth disease virus

FTIR fourier transform infrared

GFP green fluorescent protein

HDHFR human dihydropholate reductase

IPTG isopropyl-β-D-thiogalactopyranoside

ONPG ortho-nitrophenyl β-D-galactopyranoside

## Authors' contributions

EGF performed most of the experiments and prepared the final data and figures. NGM, A. Vera and AA analysed protein amounts by Western blot, RMF performed enzyme kinetics, MM performed part of optical microscopy analysis and SV part of FTIR analysis and data interpretation. A. Villaverde directed the work and prepared the manuscript.

## References

[B1] Baneyx F, Mujacic M (2004). Recombinant protein folding and misfolding in Escherichia coli. Nat Biotechnol.

[B2] Strandberg L, Enfors SO (1991). Factors influencing inclusion body formation in the production of a fused protein in Escherichia coli. Appl Environ Microbiol.

[B3] Rinas U, Tsai LB, Lyons D, Fox GM, Stearns G, Fieschko J, Fenton D, Bailey JE (1992). Cysteine to serine substitutions in basic fibroblast growth factor: effect on inclusion body formation and proteolytic susceptibility during in vitro refolding. Biotechnology (N Y ).

[B4] Baneyx F, Palumbo JL (2003). Improving heterologous protein folding via molecular chaperone and foldase co-expression. Methods Mol Biol.

[B5] Vallejo LF, Rinas U (2004). Strategies for the recovery of active proteins through refolding of bacterial inclusion body proteins. Microb Cell Fact.

[B6] Tokatlidis K, Dhurjati P, Millet J, Beguin P, Aubert JP (1991). High activity of inclusion bodies formed in Escherichia coli overproducing Clostridium thermocellum endoglucanase D. FEBS Lett.

[B7] Garcia-Fruitos E, Carrio MM, Aris A, Villaverde A (2005). Folding of a misfolding-prone beta-galactosidase in absence of DnaK. Biotechnol Bioeng.

[B8] Worrall DM, Goss NH (1989). The formation of biologically active beta-galactosidase inclusion bodies in Escherichia coli. Aust J Biotechnol.

[B9] Carrio M, Gonzalez-Montalban N, Vera A, Villaverde A, Ventura S (2005). Amyloid-like properties of bacterial inclusion bodies. J Mol Biol.

[B10] Ami D, Natalello A, Gatti-Lafranconi P, Lotti M, Doglia SM (2005). Kinetics of inclusion body formation studied in intact cells by FT-IR spectroscopy. FEBS Lett.

[B11] Sorensen HP, Mortensen KK (2005). Soluble expression of recombinant proteins in the cytoplasm of Escherichia coli. Microb Cell Fact.

[B12] de Marco A, Schroedel A (2005). Characterization of the aggregates formed during recombinant protein expression in bacteria. BMC Biochem.

[B13] Chiti F, Taddei N, Baroni F, Capanni C, Stefani M, Ramponi G, Dobson CM (2002). Kinetic partitioning of protein folding and aggregation. Nat Struct Biol.

[B14] Wright PE, Dyson HJ (1999). Intrinsically unstructured proteins: re-assessing the protein structure-function paradigm. J Mol Biol.

[B15] Waldo GS, Standish BM, Berendzen J, Terwilliger TC (1999). Rapid protein-folding assay using green fluorescent protein. Nat Biotechnol.

[B16] Carrio MM, Villaverde A (2001). Protein aggregation as bacterial inclusion bodies is reversible. FEBS Lett.

[B17] Cazorla D, Feliu JX, Villaverde A (2001). Variable specific activity of Escherichia coli beta-galactosidase in bacterial cells. Biotechnol Bioeng.

[B18] Oberg K, Chrunyk BA, Wetzel R, Fink AL (1994). Nativelike secondary structure in interleukin-1 beta inclusion bodies by attenuated total reflectance FTIR. Biochemistry.

[B19] Carrio MM, Cubarsi R, Villaverde A (2000). Fine architecture of bacterial inclusion bodies. FEBS Lett.

[B20] Sambrook J, Fritsch E, Maniatis T (1989). Molecular Cloning, A Laboratory Manual, Cold Spring Harbor Laboratory Press, Cold Spring Harbor, NY.

[B21] Feliu JX, Cubarsi R, Villaverde A (1998). Optimized release of recombinant proteins by ultrasonication of E. coli cells. Biotechnol Bioeng.

[B22] Ferraz RM, Aris A, Villaverde A (2004). Profiling the allosteric response of an engineered beta-galactosidase to its effector, anti-HIV antibody. Biochem Biophys Res Commun.

[B23] Miller JH (1972). Experiments in Molecular Genetics Cold Spring Harbor Laboratory Press, Cold Spring Harbor, NY.

[B24] Laemmli UK (1970). Cleavage of structural proteins during the assembly of the head of bacteriophage T4. Nature.

[B25] Davies JF, Delcamp TJ, Prendergast NJ, Ashford VA, Freisheim JH, Kraut J (1990). Crystal structures of recombinant human dihydrofolate reductase complexed with folate and 5-deazafolate. Biochemistry.

[B26] Sánchez de Groot N, Avilés FX, Vendrell J, Ventura S (2005). Mutagenesis of the central hydrophobic cluster in Ab42 Alzheimer's peptide. 
Simple rules to predict the aggregation propensities of polypeptides.. submitted.

